# The Arterial Supply of the Distal Part of the Pancreas

**DOI:** 10.1155/2019/5804047

**Published:** 2019-03-20

**Authors:** S. Covantev, N. Mazuruc, O. Belic

**Affiliations:** ^1^Laboratory of Allergology and Clinical Immunology, State University of Medicine and Pharmacy “Nicolae Testemitanu”, Chisinau, Moldova; ^2^Department of Human Anatomy, State University of Medicine and Pharmacy “Nicolae Testemitanu”, Chisinau, Moldova

## Abstract

The pancreatic surgery field has evolved greatly over the previous years. Nevertheless, the vascularization of the pancreas remains a difficult subject and requires further attention. The study was conducted using macroscopical dissection and corrosion cast methods. The total number of organ blocks was 72 (50 for dissection and 22 for corrosion cast). Based on the data obtained by dissection, we can distinguish three major types of vascularization of the distal pancreas. In type one, the pancreas was vascularized only by the short branches of the splenic artery and was encountered in 18 cases (36%). In type two, the pancreas was vascularized by the long and short branches of the splenic artery and was encountered in 20 cases (40%). In type three, the pancreas was vascularized only by the long branches of the splenic artery in 12 cases (24%). Compared to that, the corrosion cast method demonstrated type 1 in 8 cases (36.36%), type 2 in 10 cases (45.46%), and type 3 in 4 cases (18.18%). During the dissection, there were no arteries to the tail of the pancreas in 13 (26%) cases, one artery in 15 (30%) cases, two arteries in 19 (38%), and three arteries in three (6%) cases. The 22 corrosion cast specimens were also evaluated based on the classification of Roman Ramos and coworkers. Type I (small arcades) was in 9 (40.90%) cases, type II (small and large arcades) was in 7 (31.82%) cases, type III (large arcades) was in 5 (22.73%) cases, and type IV (straight branches) was in 1 (4.55%) case. The corrosion cast method allowed us to determine no arteries to the tail in 4 (18.18%) cases, one artery in 6 (27.27%) cases, two arteries in 10 (45.46%) cases and three arteries in two (9.09%) cases. The vascularization of the distal part of the pancreas is highly variable and should be taken into consideration during surgery.

## 1. Introduction

The field of pancreatology has evolved greatly in the last decades. This is largely due to the implementation of variety of surgical and interventional procedures for pancreatic disease as tumors, acute pancreatitis, and others. In case of pancreatic cancer, short- and long-term survival for patients has improved over time due to decreased perioperative mortality and increased use of adjuvant therapy, but the proportion of 5-year survivors still remains small [1]. This underlines the importance of early detection and more effective adjuvant therapies for patients with pancreatic cancer [2]. Taking into consideration the advancement and increased experience in the field, more complicated surgeries can be easily performed with acceptable oncologic results and decreased mortality and morbidity [3]. Pancreatic transplant is now a more widely used procedure for patients with type I diabetes mellitus with better outcomes, and the anatomy often dictates the type of procedure [4, 5]. Vascular supply of the pancreas is abundant, and the risk of hemorrhage can be high with associated mortality more than 50% [6]. All of the above-mentioned procedures require detailed understanding of the arterial supply of the pancreas. The knowledge of the types of arterial supply can improve the outcomes of the surgery and prevent possible complications. Although the field has evolved greatly, the vascularization of the pancreas remains a difficult subject and requires further attention. The rate of complications can be reduced by improving the surgical techniques and detailed knowledge of the local and regional anatomy and its surrounding structures, including collateral paths of vascularization [7].

There are numerous anatomical variations and anomalies of the pancreas that are commonly encountered during morphological and radiological assessment [8]. The classical textbook anatomy divides the pancreas into head, neck, body, and tail. In surgical practice, the pancreas is often divided into proximal anatomical segment (pancreatic head) and distal (pancreatic body and tail). Nevertheless, detailed evaluation of the vascular supply of the distal pancreas demonstrates the organ can be divided into superior and inferior body (supplied by the dorsal pancreatic artery and great pancreatic artery), inferior tail (supplied by the transverse pancreatic artery and great pancreatic artery), superior tail, and tail end (supplied by the caudate pancreatic arteries) [9].

The purpose of the study was to analyze the types of arterial supply of the body and tail of the pancreas by the long branches of the splenic artery (dorsal pancreatic artery and great pancreatic artery) and short branches of the splenic artery with an evaluation of their clinical implications.

## 2. Materials and Methods

The study was conducted using macroscopical dissection and corrosion cast methods at the Department of Human Anatomy of the State University of Medicine and Pharmacy “Nicolae Testemitanu.” The total number of organ blocks was 72 (50 for dissection and 22 for corrosion cast) and included the spleen, pancreas, and duodenum along with the vessels (part of abdominal aorta, celiac trunk, splenic artery, and superior mesenteric artery) donated to the department. The 50 organs for macroscopical dissection were preserved in 10% formaldehyde solution and then carefully dissected. The dissection was carried out to determine the main sources of vascular supply of the distal part of the pancreas. The other 22 organs were filled with autopolymerizing monomer substance of two colors (red for an artery and blue for a vein) and then preserved in an acid solution (30% hydrochloric acid) for several days until the organ was dissolved. The red monomer substance was injected with a syringe in the splenic artery at the level of the celiac trunk. The blue monomer substance was injected into the splenic vein at the level of its drainage into the portal vein. The corrosion cast method was performed to analyze the intraorganic pattern of vascular supply as well as to determine the main sources of vascular supply of the distal part of the pancreas.

The dorsal pancreatic artery was defined as a branch of the splenic artery that arises from the proximal one-third of the splenic artery and descends along the posterior margin of the pancreas where it divides into left and right branches.

The greater pancreatic artery was defined as a branch of the splenic artery that arises approximately two-thirds of the way along the splenic artery and descends along the posterior margin of the pancreas or within the substance of the pancreas itself, following the pancreatic duct.

Short pancreatic branches were defined as small vessels that arise from the splenic artery as it runs along the pancreas, supplying its body and tail.

**Figure 1:**
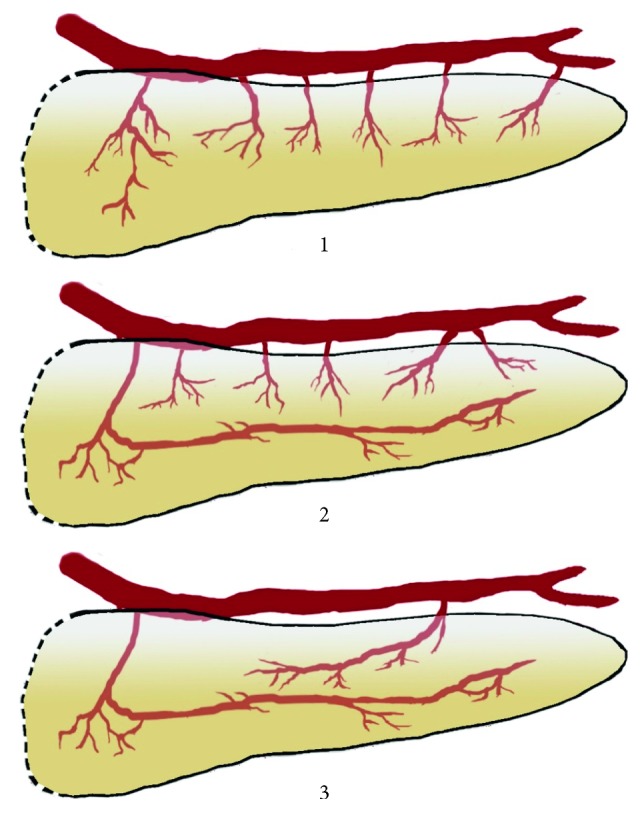
Schematic representation of the arterial supply of the pancreas based on the arteries that are involved in its arterial supply. Vascularization: 1: by the short branches of the splenic artery (type 1); 2: by the long and short branches of the splenic artery (type 2); 3: only by the long branches of the splenic artery (type 3).

**Figure 2:**
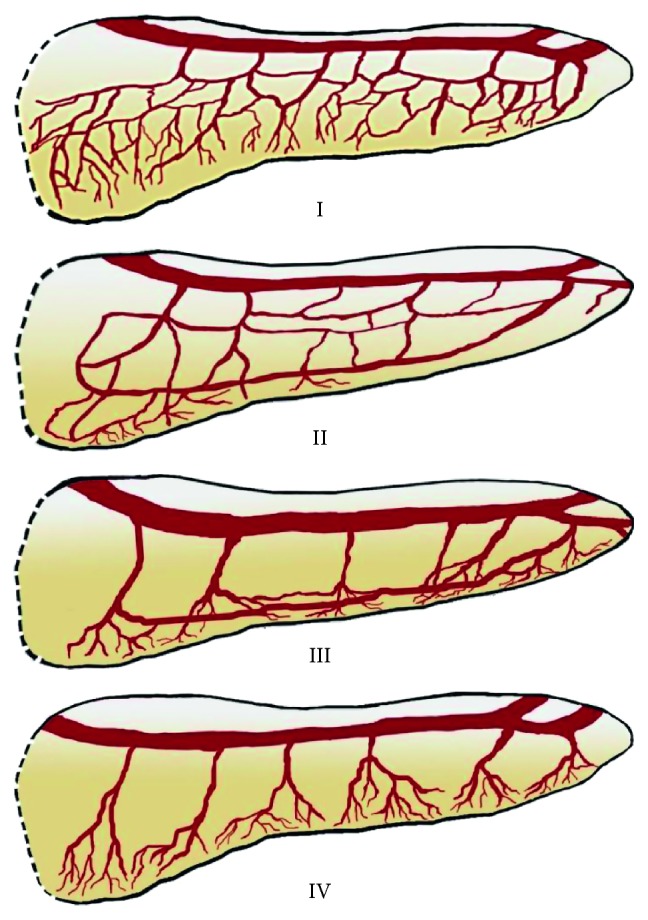
Schematic representation of the arterial supply of the pancreas by Roman Ramos and coworkers [10]. I: small arcades (type I), II: small and large arcades (type II), III: large arcades (type III), and IV: straight branches (type IV).

The vascularization of the pancreas was classified into three types: vascularization by the short branches of the splenic artery (type 1), vascularization by the long and short branches of the splenic artery (type 2), and vascularization only by the long branches of the splenic artery (type 3) (Figure 1). The short branches of the pancreases were considered branches of small caliber, which branch from the splenic artery, and after entering the parenchyma, branch to several smaller vessels. The long branches of the splenic artery were considered dorsal pancreatic artery and the great pancreatic artery. The intraorganic vascularization pattern was classified based on the four types proposed by Roman Ramos and coworkers: small arcades (type I), small and large arcades (type II), large arcades (type III), and straight branches (type IV) (Figure 2) [10].

The material for the study was acquired as a donation conforming the ethical standards of the university.

## 3. Results

**Figure 3:**
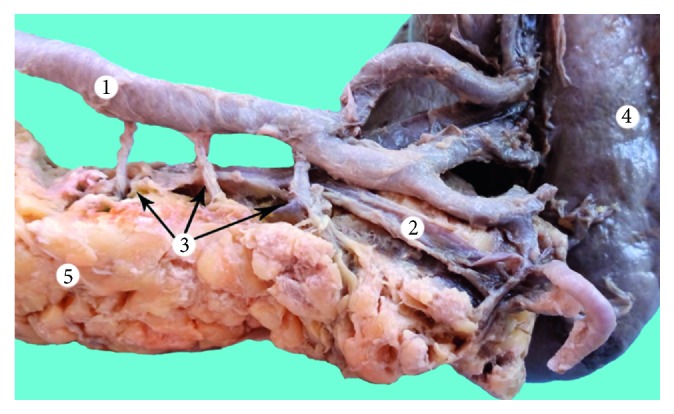
Type I vascular supply of the pancreas (dissection).1: splenic artery; 2: splenic vein; 3: short branches of the splenic artery; 4: spleen; and 5: pancreas.

**Figure 4:**
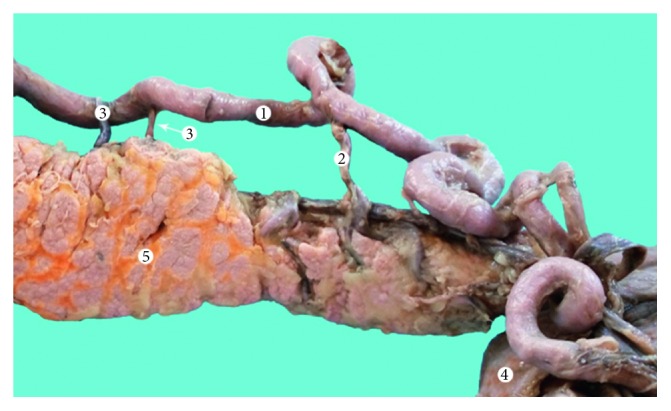
Type II vascular supply of the pancreas (dissection). 1: splenic artery; 2: dorsal pancreatic artery; 3: short branches of the splenic artery; 4: spleen; and 5: pancreas.

**Figure 5:**
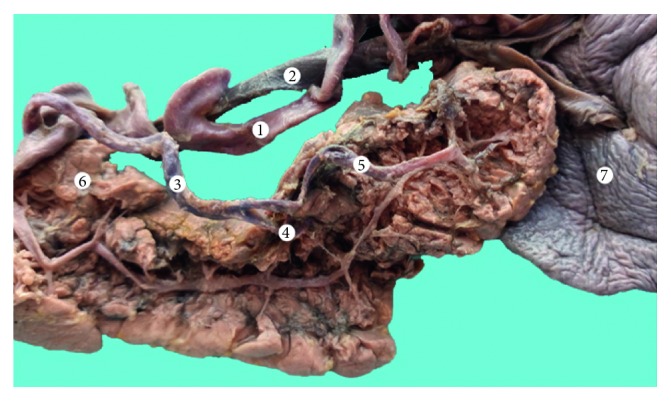
Type III vascular supply of the pancreas (dissection) 1—splenic artery; 2—splenic vein; 3—great pancreatic artery; 4—superior branch of the great pancreatic artery; 5—inferior branch of the great pancreatic artery; 6—pancreas; 7—spleen.

**Figure 6:**
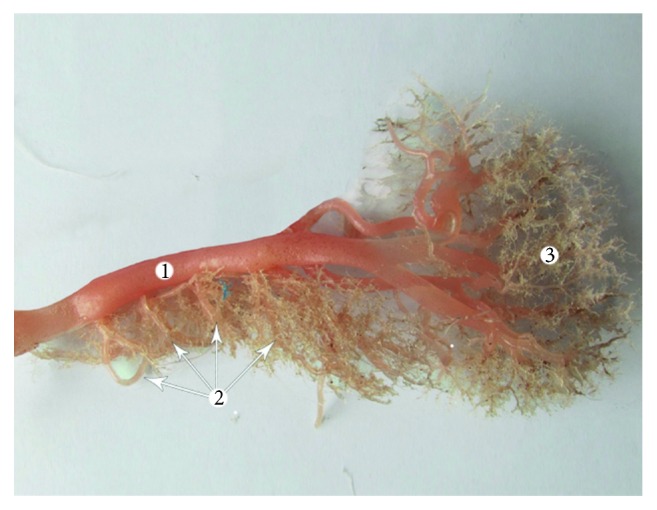
Type I vascular supply of the pancreas (corrosion cast). 1: splenic artery; 2: short branches of the splenic artery; and 3: spleen.

**Figure 7:**
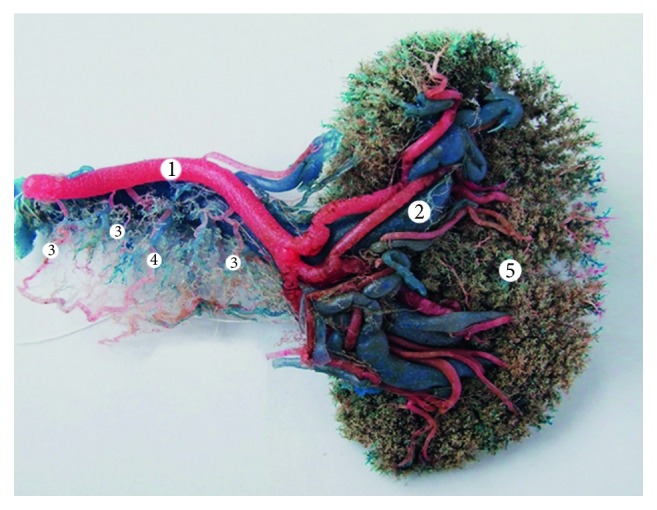
Type II vascular supply of the pancreas (corrosion cast). 1: splenic artery; 2: splenic vein; 3: short branches of the splenic artery; 4: transverse pancreatic artery; and 5: spleen.

**Figure 8:**
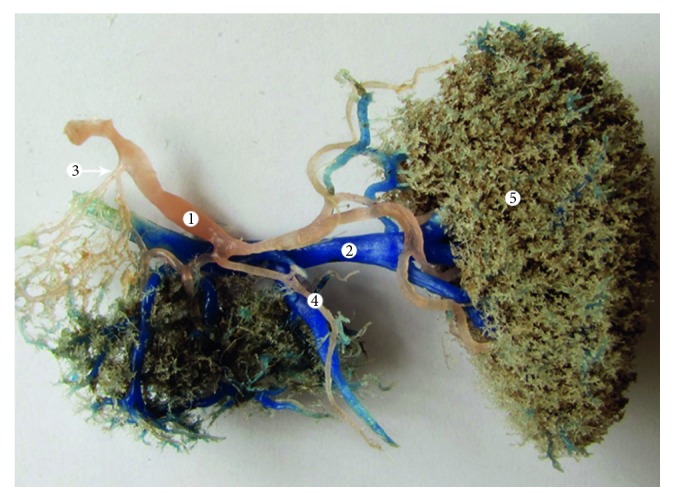
Type III vascular supply of the pancreas (corrosion cast). 1: splenic artery; 2: splenic vein; 3: superior horizontal artery of Popova; 4: caudal artery; and 5: spleen.

In type one, the pancreas is vascularized by the short branches of the splenic artery and was encountered in 18 cases (36%) (Figure 3). In type two, the pancreas is vascularized by the long and short branches of the splenic artery and was encountered in 20 cases (40%) (Figure 4). In type three, the pancreas is vascularized only by the long branches of the splenic artery in 12 cases (24%) (Figure 5). Compared to that corrosion cast method demonstrated type 1 in 8 cases (36.36%) (Figure 6), type 2 in 10 cases (45.46%) (Figure 7), and type 3 in 4 cases (18.18%) (Figure 8). The comparison of the two methods is present in Table 1.

The 22 corrosion cast specimens were also evaluated based on the classification of R. Roman Ramos and coworkers. Type I (small arcades) was in 9 (40.90%) cases, type II (small and large arcades) was in 7 (31.82%) cases, type III (large arcades) was in 5 (22.73%) cases, and type IV (straight branches) was in 1 (4.55%) case. The arteries that participated in the formation of these types of arterial supply are presented in Table 2.

Based on the results obtained from dissection, short pancreatic branches were present in 38 cases (76%) while long branches were present in 32 (64%) cases. The most frequent long branches were dorsal pancreatic artery encountered in 38 (76%) cases and the great pancreatic artery encountered in 23 (46%) cases. In 13 cases (26%), the pancreas was vascularized by both dorsal and great pancreatic arteries. The corrosion cast method demonstrated that short branches were present in 18 cases (81.81%) and long branches in 14 cases (63.63%). The transverse pancreatic artery was evaluated based on the corrosion cast specimens. It was encountered in 15 (68.18%) cases of the 22 specimens. In the majority of cases, it was a branch of the dorsal pancreatic artery (11 cases out of 15; 73.33%).

We also noticed some of the less frequent variations of development like the superior horizontal artery of Popova. During the dissection, we encounter superior horizontal artery of Popova in two (5%) cases and additional one case (4.54%) from the group with corrosion casts (Figure 8).

During the dissection, we encountered no arteries to the tail of the pancreas in 13 (26%) cases, one artery in 15 (30%) cases, two arteries in 19 (38%), and three arteries in 3 (6%) cases. The corrosion cast method allowed us to determine no arteries to the tail in 4 (18.18%) cases, one artery in 6 (27.27%) cases, two arteries in 10 (45.46%) cases, and three arteries in 2 (9.09%) cases. The comparison of the two methods is present in Table 3.

## 4. Discussion

The vascularization of the pancreas is a complicated subject, and over the years, there were several propositions on how to classify the blood supply of the body and tail of the pancreas.

Based on our results in the majority of cases, the pancreas is vascularized by short and long branches (type 2, 40–45.5%). The pancreas is vascularized by only short branches (type 1, 36–36.36%) less frequently. The rarest type of vascularization is the vascularization by only long branches (type 3, 18.18–24%).

There are other ways to classify the vascularization of the pancreas. Roman Ramos and coworkers describe 4 types based on intraorganic distribution: small arcades (type I), small and big arcades (type II), large arcades (type III), and straight branches (type IV) [10]. This classification is easier to use in case of corrosion cast or radiological studies. It also underlines an important point. There are cases when the pancreas does not have large anastomosis between its branches. This allows an easier resection and less blood loss (type IV). However, this type is seen rare (one case, 4.54%). This finding supports the idea that, in the majority of cases, it is difficult to consider pancreas as a segmental organ. Nevertheless, in rare cases, there may be some degree segmental structure of this gland. It also can be useful in case of enucleation of small benign tumors and intermediate pancreatomy. In these cases, the presence of less prominent anastomoses can be used as an advantage as it allows less blood loss and less possibility of necrosis. Therefore, in the majority of cases, the vascularization of the pancreas is abundant with anastomoses (types I and II). These two types are predominant in our study (type I: 40.9%, type II: 31.82%). Type III represents some degree and intermediate type and is seen less frequently (22.73%). In this case, the anastomoses are present in the inferior region of the pancreas. The loops typically are continuations of large branches of the splenic artery (dorsal pancreatic artery and great pancreatic artery), whereas the short branches typically supply only a small region of the gland probably due to their diameter.

The idea of a network of loops which have different forms and sizes is supported by other studies, and the networks tend to be more prominent in newborns and decrease with age (after 35 years) due to involution processes [10, 11]. A more extensive evaluation of the vascular supply using angiography is presented by Okahara and coworkers, who divide the distal pancreas based on its vascular supply into superior and inferior body (supplied by the dorsal pancreatic artery and great pancreatic artery), inferior tail (supplied by the transverse pancreatic artery and great pancreatic artery), superior tail, and tail end (supplied by the caudate pancreatic arteries) [9].

It is important to mention that the results of the study often depend on the method. The corrosion cast method is considered a more precise method to study anatomy compared to manual dissection. It also gives a more detailed representation of the intraorganic distribution of the vessels and allows determining zones where the vascularization is not so abundant, thus favoring resection. On the other hand, the quality of the corrosion cast method depends on the type of materials used and does not always show arteries of small calibers or those that are blocked. Anatomical dissection is method that is largely based on the skills of the dissector. Finally, the anatomy often depends on the region of the world and ethnical group. The results obtained in some ethnical groups are not always applicable to other.

All of the pancreatic arteries that were studied deserve separate attention. According to angiography, the great pancreatic artery is found in 73.1%–82% of cases [12, 13]. The number of arteries is also variable: one artery in 26–60%, two arteries in 20–33%, three in 2–6%, and four in 2–48% (depending on the author and the study method) [13, 14]. During anatomical dissection, the artery can be found in 64.7–98% of cases [15]. However, some authors report that it is found less frequently—5.21–10% of the cases [16, 17]. Although it is called great, its diameter is usually smaller than dorsal pancreatic artery but exceptions can also occur [18].

The superior horizontal pancreatic artery of Popova is considered a variant of the great pancreatic artery and runs along the superior border of the pancreatic body and tail. It is seen in 25.93% of cases [19]. We report a lower incidence of this artery (5% of the dissected specimens and 4.54% of the corrosion cast group).

The detection rates for dorsal pancreatic artery vary (65.4–94%) [12, 20, 21]. During anatomical dissection, the dorsal pancreatic artery can be identified in 88.8% [22]. It usually begins from the splenic artery, common hepatic artery, superior mesenteric artery, or celiac trunk [23]. The dorsal pancreatic artery is important during pancreatic transplant. Organ procurement errors account for almost 20% of discarded pancreatic allografts. And, in case of pancreatic transplant, the dorsal pancreatic artery provides together with the splenic artery the main blood supply to the pancreatic tail [24]. It also often supplies the majority of the body of the pancreas along with the duct, and thus, its damage can cause ischemia and necrosis with serious complications [25]. Based on the origin of the artery, Fiedor and coworkers classify the vascularization in five forms: the dorsal pancreatic artery branched off from the splenic artery (form I); the dorsal pancreatic artery branched directly off from the celiac trunk (form II); the dorsal pancreatic artery branched off from the common hepatic artery (form III); the dorsal pancreatic artery was a branch of the superior mesenteric artery (form IV); and the dorsal pancreatic artery branched off from the gastroduodenal artery (form V). Clinically, this classification is dictated by the fact that variations of the dorsal pancreatic artery influence the arterial vascularization of the pancreas, the number of collaterals, and anastomoses with other arterial vessels of various segments of the organ [25]. This classification can explain why in some cases the dorsal pancreatic artery is not always present in our study.

Finally, although we did not pay special attention to the arteries that run transversely in the pancreas (transverse pancreatic artery and superior transverse pancreatic artery), they still should be discussed. Based on the data from the literature, the superior transverse pancreatic artery is seen in 63.2% of cases and runs along the superior ventral side of the head of the pancreas in 79.2% of cases. It is formed between the gastroduodenal and dorsal pancreatic arteries in 39.5% of cases. The transverse pancreatic artery runs along the inferior surface of the body and tail of the pancreas and anastomoses with the dorsal pancreatic artery or the great pancreatic artery [26].

The pattern of anastomosis between the pancreas tail and the spleen can impact surgery in cases of splenic trauma, favoring splenic preservation [27]. Сaudal pancreatic arteries originate from the splenic artery at its distal third (55.5–70%), from its terminal inferior branch (30%) or the left gastroepiploic artery (9.3%) [25, 27]. The incidence of caudal pancreatic arteries varies in 39–96.2% [12, 21]. The number of the arteries is also variable (in medium approximately 3). In our study, the tail of the pancreas is supplied by two arteries (38–45.5%), followed by one artery (27.24–30%) and no arteries at all (18.6–26%) depending on the method. Vascularization of the tail of the pancreas by three arteries can be found in rare cases (6–9.08%). Ebner and coworkers report five basic types of relations between caudal arteries and other vessels: Type I: the tail is supplied exclusively by caudal arteries. Type II: at least one caudal artery anastomoses with the vessels of the corpus. Type III: the cauda is supplied both by the caudal arteries and by vessels of the corpus (nonanastomosing). Type IV: combined forms of blood supply by caudal arteries and corpus arteries by way of anastomoses and nonanastomosing vessels are found. Type V: the cauda is supplied exclusively be vessels stemming from the corpus [28]. Finally, tail morphology can be different and have complicated relationship with the vascular supply [29].

When taking into consideration the vascularization pattern of the pancreas, it should be noted that pancreatic body and tail included the dorsal pancreatic artery alone (50%), combined dorsal pancreatic artery and great pancreatic artery (21.6%), great pancreatic artery alone (15.7%), and transverse pancreatic artery (10.8%) [30]. The blood supply to the body and tail often has significant anastomoses with the head of the pancreas [31]. Other authors state that, in 42.1%, the tail has an autonomous vascular supply [25]. Our dissection study demonstrates the dorsal pancreatic artery encountered in 76% cases and the great pancreatic artery encountered in 46% cases. In only 26% of cases, the pancreas was vascularized by both dorsal and great pancreatic arteries.

Arterial supply in this region is important for the interventional procedures on the pancreas and spleen since both splenic and pancreatic pathology can result in severe complications such as pancreatic necrosis after splenic artery embolization [32]. It is also important to study the vascularization of these organs together since its relationship is important in clinical practice. Acute pancreatitis can also lead to splenic artery pseudoaneurysm, splenic vein thrombosis, bleeding, and other [33, 34]. Acute bleeding is a rare but frequently fatal complication of pancreatitis and may occur from eroded vessels or pseudoaneurysms [35]. The mortality of this complication may be up to 50% depending on the vessel and severity [36].

The types of vascularization and developmental variations of vascular supply should be considered during surgical and interventional procedures such as selective catheterization and for a limited operative intervention, pancreatic transplant and other [5, 31].

One of the major limitations of the study is that the vessels, which run transversely in the pancreas, have not been analyzed in the study whereas they play a major role in its vascular supply. The other limitations of the study include relatively low number of specimens and the fact that sex and age have not been taken into consideration. These limitations should be taken into account in the future studies.

## 5. Conclusions

The vascularization of the distal part of the pancreas is highly variable. The course and number of the arteries as well as the type of vascularization should be taken into consideration during surgical procedures since they determine the volume and type of surgical procedures and can prevent intraoperative and postoperative surgical complications. Over the years, several classifications have been proposed to better understand the types of blood supply. These observations demonstrate that, in some cases, the gland has a segmental structure, which allows introducing organ-sparing procedures in some cases as well as improvement of the organ-harvesting techniques. Some types of intraorganic vascular distribution make it easier to perform organ-sparing procedures.

## Figures and Tables

**Table 1:** Types of pancreas vascularization and their frequency.

	I type	II type	III type	Total
Dissection, *n* (%)	18 (36.0)	20 (40.0)	12 (24.0)	50 (100)
Corrosion cast, *n* (%)	8 (36.36)	10 (45.46)	4 (18.18)	22 (100)

**Table 2:** Arteries that participated in the formation of vascular patterns.

Type	Small pancreatic branches	Dorsal pancreatic artery	Greater pancreatic artery
I (small arcades), *n* (%)	9 (100)	2 (22.22)	3 (33.33)
II (small and large arcades), *n* (%)	5 (71.42)	6 (85.71)	5 (71.42)
III (large arcades), *n* (%)	3 (60.0)	5 (100.0)	4 (80.0)
IV (straight branches), *n* (%)	1 (100)	—	—

**Table 3:** Number of arteries that vascularize the tail of the pancreas.

	No arteries	One artery	Two arteries	Three arteries	Total
Dissection, *n* (%)	13 (26)	15 (30)	19 (38)	3 (6)	50 (100)
Corrosion cast, *n* (%)	4 (18.18)	6 (27.27)	10 (45.46)	2 (9.09)	22 (100)

## Data Availability

The data used to support the findings of this study are available from the corresponding author upon request.
